# Alterations of Hematologic and Hematopoietic Parameters in Mice Exposed to Pulsed Electromagnetic Field

**DOI:** 10.1155/2019/3628956

**Published:** 2019-03-24

**Authors:** Kangchu Li, Zenghui Teng, Zhaohui Liu, Yuxing Zhang, Kaiping Long, Qimei Liao, Ansheng Ni, Guirong Ding, Shirong Ma

**Affiliations:** ^1^Medical College, Xijing University, Xi'an, 710123 Shaanxi, China; ^2^Department of Pharmacology, School of Pharmacy, Southwest Medical University, Luzhou, 646000 Sichuan, China; ^3^Department of Rehabilitative Physiotherapy of Tangdu Hospital, Fourth Military Medical University, Xi'an, 710038 Shaanxi, China; ^4^Department of Radiation Biology, School of Preventive Medicine, Fourth Military Medical University, Xi'an, 710032 Shaanxi, China; ^5^Department of Pathology, School of Basic Medicine, Fourth Military Medical University, Xi'an, 710032 Shaanxi, China

## Abstract

Effects of pulsed electromagnetic field (PEMF) on hematology and hematopoiesis might vary with different PEMF parameters. The purpose of this study was to evaluate the possible effects of PEMF exposure at different pulses on hematologic and hematopoietic parameters in mice. Groups of male BALB/c mice were whole body exposed or were sham exposed (control) to PEMF at 100, 1000, and 10000 pulses. After PEMF exposure, blood samples and bone marrow cells of mice were collected for hematologic examinations, bone marrow nucleated cell counting, colony-forming units of granulocyte-macrophage (CFU-GM) colony assay, and serum granulocyte-macrophage colony-stimulating factor (GM-CSF) assay. Compared with the control group, white blood cells (WBC) and lymphocytes (LYM) in the 100 and 1000 pulses exposed groups were significantly increased but not changed in the 10000 pulses exposed group. Red blood cells (RBC), hemoglobin (HGB), and platelets (PLT) were not changed in all exposed groups. There was no significant difference in mouse bone marrow nucleated cell number between the control group and each exposed group 7 days after PEMF exposure. The CFU-GM clone number of bone marrow cells and serum GM-CSF level were significantly increased in the 100 and 1000 pulses exposed group but not changed in the 10000 pulses exposed group. Our results indicated that the PEMF exposure at fewer pulses may induce statistically significant alterations in some hematologic and hematopoietic parameters of mice but no changes can be found in the more pulses PEMF-exposed groups.

## 1. Introduction

With the development and application of electromagnetic technology, the potential health risk of electromagnetic exposure received more and more concerns. It was reported that pulsed electromagnetic field (PEMF) led to some biological effects on varied parts of the body, such as the gingiva [1], kidney [2], and brain [3]. Moreover, epidemiological studies showed a correlation between environmental electromagnetic fields and leukemia [4–7]. Many studies focused on the effects of electromagnetic fields on hematology and hematopoiesis. Some reported that electromagnetic fields had effects on hematology and hematopoiesis [8–11]. But on the other hand, some reported the negative results [12–17]. So far, there was no agreement available. According to the previous studies, effects of PEMF on hematology and hematopoiesis might vary with different PEMF parameters such as field intensity and frequency.

The PEMF applied in this study was a special electromagnetic field with high-voltage pulses and an extremely fast rise time. It was extensively used in military campaigns, security screening, medical applications, and many other fields. The increasing exposed opportunity of this kind of PEMF has raised concerns about possible implications to human health. Results of the previous studies in our lab showed that the PEMF we used caused changes in the blood-brain barrier [18, 19], embryogenesis [20], and bone formation of osteoblasts [21]. However, effects of such kind of PEMF on hematology and hematopoiesis were not clear. In order to answer this question, the current study was designed to evaluate the possible effects of PEMF exposure at different pulses on hematologic and hematopoietic parameters in the blood and bone marrow of BALB/c mice.

## 2. Materials and Methods

### 2.1. Experimental Animals

Forty healthy male BALB/c mice (20 ± 2 g), 8–10 weeks old, were obtained from the Fourth Military Medical University (FMMU) Experimental Animal Center (Xi'an, China). All studies were performed with the approval of the experimental animal care committee of the Fourth Military Medical University. Animals were allowed free access to laboratory chaw and water. The ambient temperature and relative humidity of the animal room were 21 ± 1°C and 60 ± 7%, respectively. The room was illuminated with artificial light for 12 hours daily and was dark for 12 hours at night. Animals were randomly divided into four groups (*n* = 10): the 100, 1000, and 10000 pulses exposed group and the sham-exposed (control) group.

### 2.2. PEMF Exposure

PEMF (electric field intensity of 100 kV/m, repetition frequency of 50 Hz) was generated by a spark gap pulse generator and transmitted to the animal platform. A special plastic box was placed on the animal platform and animals were able to move freely in the box. Four exposed protocols were used: 100 pulses, 1000 pulses, 10000 pulses, and sham exposure (control). The exposure time is 2 seconds, 20 seconds, and 200 seconds, respectively. The groups of mice were exposed to PEMF or were sham exposed at the same time, and the temperature control measurements showed that there were no changes in temperature during the exposure.

### 2.3. Hematologic Analysis

Blood samples were collected and placed into 3-4 mL tubes anticoagulated by EDTA-2K for hematologic study 7 days after PEMF exposure. White blood cells (WBC), red blood cells (RBC), hemoglobin (HGB), platelets (PLT), and lymphocytes (LYM) were determined using a blood counter (K-4500, Sysmex, Japan).

### 2.4. Bone Marrow Nucleated Cell Counting

The animals were slaughtered 7 days after PEMF exposure, and the bone marrow cells were flushed from the left femur with 1 mL of RPMI-1640 medium (Gibco) supplemented with 10% heat-inactivated fetal bovine serum (Hyclone Labs, Logan, UT). The number of nucleated cells in the bone marrow suspensions was determined microscopically by cell counting with the hemocytometer.

### 2.5. CFU-GM Assay

The bone marrow cells of each group were aseptically collected from the right femur of mouse 7 days after PEMF exposure. Briefly, the plug of the marrow was gently extruded into a sterile plastic tube by 1 mL of RPMI-1640 medium (Gibco) injected through the femur. The worm-like marrow plug was then converted into a dispersed cell suspension in 3 mL of RPMI medium by gently aspirating the suspension up and down 20 times using a sterile 5 mL pipette. Colony-forming units of granulocyte-macrophage (CFU-GM) colony assays with cell suspensions from femoral marrow were performed in 1 mL agar cultures in 35 mm Petri dishes using 1 × 10^5^ marrow cells per culture. The medium used was RPMI-1640 containing 30% horse serum and 0.3% of agar. The cultures were incubated for 7 days in a fully humidified atmosphere of 5% CO_2_, and colony formation (clones >50 cells) was scored at 40-fold magnification using a dissection microscope.

### 2.6. Serum GM-CSF Assay

Serum samples were collected 7 days after PEMF exposure, and the levels of granulocyte-macrophage colony-stimulating factor (GM-CSF) were measured by enzyme-linked immunosorbent assay (ELISA) using commercially available kits. Studies were performed according to the manufacturer's instructions. Optical density was read on an automated microplate photometer.

### 2.7. Statistical Analysis

Comparisons of data among all groups were analyzed using one-way ANOVA test. Statistical analyses were performed with the SPSS 13.0 software. All data were presented as mean ± standard deviation (SD). Differences were considered statistically significant at *P* < 0.05.

## 3. Results

### 3.1. Changes of Hematologic Parameters in Mice Exposed to PEMF at Different Pulses

**Figure 1:**
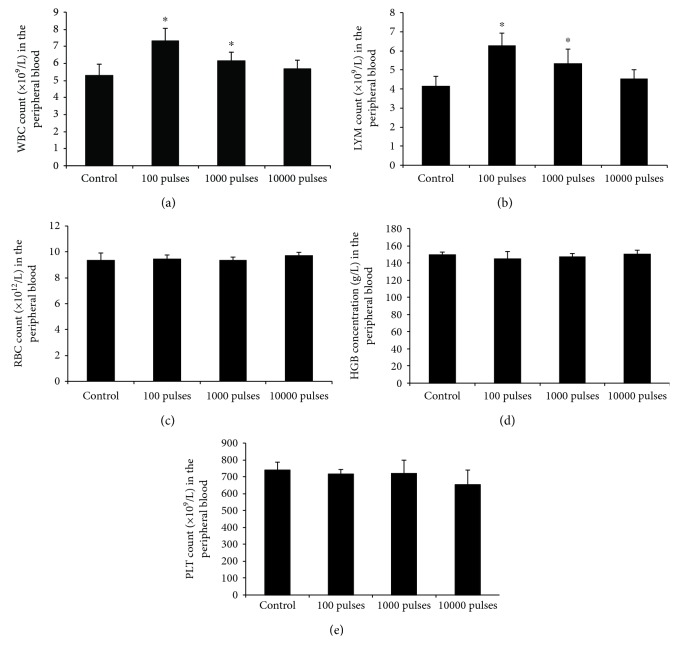
Changes of hematologic parameters in mice after exposed to PEMF at different pulses. Blood samples were collected and the hematologic parameters including WBC (a), LYM (b), RBC (c), HGB (d), and PLT (e) were examined. All data were presented as mean ± SD. ^∗^*P* < 0.05 vs. control.

Statistically significant increases in peripheral blood WBC and LYM occurred in animals of the 100 and 1000 pulses exposed groups compared with the control groups (*P* < 0.05) but not in animals of the 10000 pulses exposed group 7 days after PEMF exposure. WBC and LYM were relatively lower in the 1000 pulses exposed groups than those in the 100 pulses exposure groups (Figures 1(a) and 1(b)). There were no statistically significant differences in RBC, HGB, or PLT of all exposed groups compared with the control groups (Figures 1(c)–1(e)).

### 3.2. Changes of Bone Marrow Nucleated Cell Number in Mice Exposed to PEMF at Different Pulses

**Figure 2:**
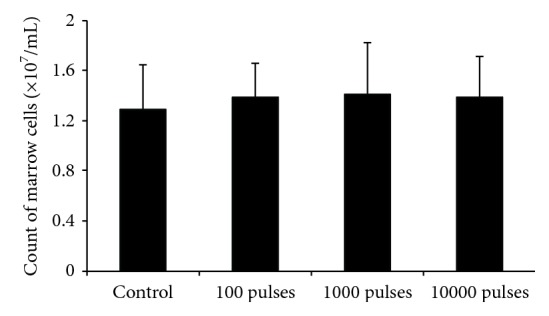
Changes of bone marrow nucleated cell number in mice after exposed to PEMF at different pulses. The bone marrow cells were flushed from the left femur with 1 mL of RPMI-1640 medium supplemented with 10% heat-inactivated fetal bovine serum. The number of nucleated cells was determined by cell counting. All data were presented as mean ± SD.

To evaluate the effects of PEMF at different pulses on hematopoietic parameters, we first observed the response of bone marrow nucleated cell number.  Figure 2 shows that 7 days after the animals were exposed to PEMF at 100, 1000, or 10000 pulses, mouse bone marrow nucleated cell number was not significantly changed compared with the control group (*P* > 0.05).

### 3.3. Changes of CFU-GM in Mice Exposed to PEMF at Different Pulses

**Figure 3:**
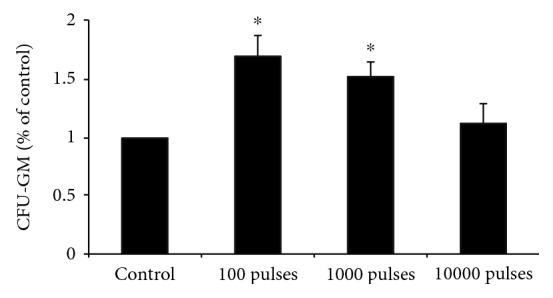
Changes of CFU-GM in mice after exposed to PEMF at different pulses. The clonogenicity was expressed as a percentage relative to the number of colonies counted in control dishes which was considered 100% of clonogenicity. All data were presented as mean ± SD. ^∗^*P* < 0.05 vs. control.

In consistent with the data of peripheral blood WBC and LYM, the CFU-GM clone number of bone marrow cells in all the PEMF-exposed groups was higher than that in the control group and decreased with PEMF exposure pulses getting more. The CFU-GM clone number was significantly increased in animals of the 100 and 1000 pulses exposed group compared with the control group (*P* < 0.05), but no statistically changes were found in animals of the 10000 pulses exposed group 7 days after PEMF exposure (Figure 3).

### 3.4. Changes of GM-CSF Serum Level in Mice Exposed to PEMF at Different Pulses

**Figure 4:**
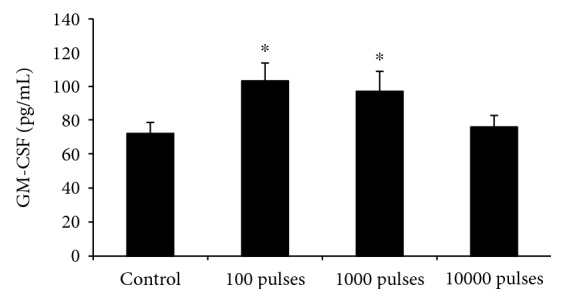
Changes of GM-CSF level in mouse serum after exposed to PEMF at different pulses. Blood samples were collected and the level of GM-CSF was assessed using the standard ELISA method. All data were presented as mean ± SD. ^∗^*P* < 0.05 vs. control.

We further examined the GM-CSF level in serum after the mice were exposed to PEMF at different pulses. As shown in Figure 4, the serum GM-CSF level in either the 100 or 1000 pulses exposed group was significantly higher than that in the control group (*P* < 0.05). However, there was no statistically significant difference in the mouse serum GM-CSF level between the 10000 pulses exposed group and the control group (*P* > 0.05).

## 4. Discussion

In the present study, we found that the applied PEMF exposure may increase WBC and LYM at fewer pulses rather than at more pulses. These findings were in agreement with those from other experimental studies. Ragan et al. found that significant increases were occasionally seen in WBC and LYM from the PEMF-exposed groups, but were not consistently observed [22]. Bonhomme-Faivre et al. also reported that the effects of electromagnetic field on RBC changed with different exposure duration [23]. However, there were some negative [24–26] or even opposite [27–29] results available. The reason of the contradiction was possibly due to the difference in electromagnetic fields used in different studies.

As for RBC, HGB, and PLT, we did not find any significant difference between the control group and each PEMF-exposed group, indicating that RBC, HGB, and PLT were relatively less sensitive to the applied PEMF compared to WBC and LYM. Many previous studies also reported that electromagnetic fields had no significant effects on RBC, HGB, and PLT. Selmaoui et al. reported that no significant differences were observed between sham-exposed (control) and exposed men for RBC, HGB, and PLT [25]. The results of Wong et al. showed no statistically significant differences from controls for RBC and HGB [26]. Seto et al. also noted that after electromagnetic field exposure, none of the red cell parameters differed significantly, although WBC and LYM were significantly changed in PEMF-exposed subjects [29], which is partially consistent with our results. In contrast to the previous studies, we used PEMF with different parameters (electric field intensity of 100 kV/m) but found the similar results on RBC, HGB, and PLT. Our data further confirmed the low sensitivity of RBC, HGB, and PLT in peripheral blood to PEMF exposure.

Hematopoiesis is a continuous process, where mature blood cells are replaced by the proliferation and differentiation of more primitive progenitor and stem cells. In order to find the reason why WBC and LYM can be affected by PEMF exposure at fewer pulses, the nucleated cell number and the CFU-GM colony formation of bone marrow cells were evaluated in current study. In the evaluation of effects of PEMF at different pulses on bone marrow nucleated cell number, we could not find a significant difference between each two groups including the control group. This data indicated that 100 kV/m PEMF exposure at 100, 1000, or 10000 pulses could not cause significant changes in mouse bone marrow nucleated cell number.

According to previous studies, the effects of electromagnetic field on CFU-GM colony formation of hematopoietic progenitor cells were also controversial. Some previous studies demonstrated that CFU-GM clone-forming properties of hematopoietic progenitor cells were affected by electromagnetic field [23, 30, 31]. However, some studies found no significant alterations of clonogenic efficiency on hemopoietic cells after electromagnetic field exposure [32–34]. Until now, no agreement on this issue was available. In this study, we found that PEMF exposure at fewer pulses (100 and 1000 pulses) stimulated CFU-GM colony formation of bone marrow cells of mice while PEMF exposure at more pulses (10000 pulses) could not affect CFU-GM colony formation. This was in accordance with the hematologic results in this study, indicating that the promotive effects of PEMF exposure at fewer pulses on WBC and LYM may be the result from the increase of CFU-GM colony formation.

GM-CSF is a cytokine that functions as a WBC growth factor which stimulates stem cells to produce granulocytes and monocytes. So far, there were little reports about the effects of electromagnetic fields on GM-CSF level. Chang et al. reported that PEMF with different intensities could regulate the macrophage colony-stimulating factor (M-CSF) concentrations in marrow culture system [35], but they did not focus on the relationship between M-CSF level and hematopoiesis. Our present data showed that PEMF exposure at fewer pulses could increase the GM-CSF serum level, while the stimulant effects could not be seen after PEMF exposure at more pulses. This trend is accordant with the effects on WBC, LYM, and CFU-GM colony formation, indicating the possible correlation between them.

In conclusion, our results showed that the applied PEMF exposure at fewer pulses stimulated some hematologic and hematopoietic parameters of mice, but the stimulant effects decreased with the PEMF-exposed pulses getting more. There were no stimulant effects when the PEMF-exposed pulses reached 10000. It indicated that PEMF exposure at more pulses (>10000) may possibly repress those hematologic and hematopoietic parameters. However, the answer to this question awaits further studies.

## Data Availability

The data used to support the findings of this study are included within the article.
